# PEG Surface Modification of ZnO Nanoparticles Improves Ductility and Absorption-Dominant EMI Shielding in PLA/PBS/Carbon Fiber Composites

**DOI:** 10.3390/polym18192388

**Published:** 2026-09-30

**Authors:** Necla Altin, Saeid Darvishi, Bedriye Ucpinar, Alp Oral Salman, Ayse Aytac

**Affiliations:** 1 Department of Chemical Engineering, Faculty of Engineering, Kocaeli University, 41001 Kocaeli, Türkiye; necla.altin@kocaeli.edu.tr (N.A.); aaytac@kocaeli.edu.tr (A.A.); 2 Department of Chemical Engineering, Faculty of Engineering, Yeditepe University, 34755 Istanbul, Türkiye; bedriye.ucpinar@yeditepe.edu.tr; 3 Department of Electronics and Communication Engineering, Faculty of Engineering, Kocaeli University, 41001 Kocaeli, Türkiye; oral.salman@kocaeli.edu.tr; 4 Department of Polymer Science and Technology, Kocaeli University, 41001 Kocaeli, Türkiye

**Keywords:** poly(lactic acid), poly(butylene succinate), PEG-coated ZnO nanoparticles, carbon fibre, electromagnetic interference shielding, steric stabilization, biodegradable polymer matrix, absorption-dominant shielding

## Abstract

Composites based on biodegradable polymers are attractive replacements for metallic electromagnetic interference (EMI) shields, but their performance is limited by nanofiller agglomeration. In poly(lactic acid)/poly(butylene succinate) (PLA/PBS) composites reinforced with carbon fiber (CF), unmodified ZnO nanoparticles agglomerate above 1 wt %, severely embrittling the material. In this study, ZnO nanoparticles were coated with poly(ethylene glycol) (PEG, 8 kDa) by solution adsorption and incorporated at 0.5–2 wt % into PLA/PBS (80/20) containing 20 wt % CF. FTIR showed a +14 cm^−1^ shift in the ether C–O–C band (1093 → 1107 cm^−1^), consistent with adsorption of PEG on the ZnO surface. Fracture surfaces showed uniform sub-micron dispersion, in contrast to the dense interfacial clusters formed by unmodified ZnO at the same loading. At 1 wt % PZ, the composites combined 76.3 MPa tensile strength and 2.35% strain with 71 dB X-band shielding effectiveness, more than three times the 20 dB commercial requirement. The highest shielding, 89 dB, was reached at 1.5 wt %, with absorption contributing more than 90% of the total. Unmodified ZnO required about five times this loading to reach comparable shielding.

## 1. Introduction

The proliferation of high-frequency electronics and the deployment of 5G/6G communication systems have intensified electromagnetic interference (EMI), which degrades the performance of sensitive devices and is most consequential in the X-band (8–12 GHz) [1]. Conventional shields based on aluminum, copper, and nickel achieve high shielding effectiveness but are heavy, corrosion-prone, difficult to process and reflection-dominant, so that the attenuated energy is returned to the environment as secondary EMI rather than dissipated [2]. Polymer-based composites offer a lightweight, melt-processable and tunable alternative in which the shielding response can be engineered through filler selection [3]. When the host polymer is additionally biodegradable, the same material addresses a second and independent environmental burden, namely, the accumulation of persistent petroleum-derived plastics in electronic waste streams [4].

The application that motivates this work is the injection-molded enclosure of short-lifecycle electronics including housings, covers and internal shielding partitions for consumer wearables, IoT and 5G small-cell modules, and single-use medical devices. Equipment of this class is manufactured in high volume and discarded within a few years, and it is a principal contributor to the 62 Mt of electronic waste generated worldwide in 2022, of which approximately 17 Mt was plastic [5]. The shielding requirement for a part of this kind is modest by laboratory standards: 20 dB is widely taken as the threshold for commercial use, and 30–40 dB is sufficient for sensitive or regulated equipment [6,7,8]. What distinguishes the requirement is that it must be satisfied together with three further constraints. First, the attenuation should be absorption- rather than reflection-dominant, because energy reflected at an enclosure wall is returned to the device interior and to the surroundings as secondary EMI rather than dissipated [2,9]. Second, the compound must survive being made into a part and assembled into a product, which sets a lower bound on ductility, since brittleness is the principal barrier to the use of PLA-based materials in molded and mechanically assembled components [10]. Third, the material must retain its stiffness above ambient temperature, the low heat-deflection temperature of PLA being a further recognized limitation for durable-goods applications [10,11]. These three constraints, rather than a maximum attainable shielding effectiveness, define the target against which the compositions reported here are assessed, and they set the scope of the characterization presented below.

Among candidate biodegradable matrices, poly(lactic acid) (PLA) and poly(butylene succinate) (PBS) are particularly attractive. PLA is a renewable, biocompatible polyester with high stiffness and moderate thermal stability, but its brittleness (elongation at break below 6%), low impact resistance and slow crystallization limit its standalone structural use [10,11]. Blending PLA with the tougher, more flexible PBS yields partially miscible systems with synergistic mechanical performance while preserving biodegradability [10,12]. An 80/20 PLA/PBS ratio offers an effective balance between rigidity and ductility and is adopted here [13].

A percolating conductive network is required to impart shielding into an otherwise electromagnetically transparent matrix. Carbon fiber (CF) is an effective primary reinforcement: its high conductivity and modulus establish a conductive backbone that attenuates electromagnetic waves through both reflection and absorption, and a 20 wt % loading is widely regarded as near-optimal for forming an efficient network without the processing penalties of excessive fiber content [14,15,16]. The addition of 20 wt % CF alone raises the total shielding effectiveness (SE_T_) of PLA/PBS from 1.7 to 30.7 dB [14], and comparable behaviour has been reported for short-CF-reinforced PLA systems [17]. Semiconducting ZnO nanoparticles act as a complementary dielectric filler, enhancing attenuation through interfacial polarization, dipole relaxation and the formation of dielectric bridges within the CF network that amplify multiple internal scattering [18]. Hybrid PLA/PBS/CF/ZnO composites have reached SE_T_ values of 70–85 dB with absorption accounting for more than 80% of the total, which is desirable because absorption-dominant shielding suppresses secondary reflection [14].

This performance is nonetheless constrained by a dispersion limitation. Above approximately 1 wt %, unmodified ZnO nanoparticles agglomerate in the PLA/PBS/CF system, with three coupled consequences: the CF-supported network is disrupted, the composite embrittles to the point that elongation at break falls to 0.06% at 7 wt %, and SE_T_ varies non-monotonically, dipping near 3 wt % before recovering at higher loadings [14]. The origin of this behaviour is the high surface energy and the polar surface hydroxyl population of bare ZnO, which drive van der Waals and hydrogen-bond-mediated aggregation and render the particles thermodynamically incompatible with a hydrophobic polyester matrix. An elongation at break of this order lies below the ductility a molded and mechanically assembled part requires, so the dispersion limitation is a manufacturability constraint as much as a mechanical one. The same limitation is reported broadly for ZnO-filled PLA and PLA/PBS systems, where it has been studied mainly in the context of ultraviolet resistance, antimicrobial action and gas barrier performance rather than shielding [19,20,21,22].

Coating nanoparticles with a polymeric surfactant is an established route to mitigate agglomeration, and poly(ethylene glycol) (PEG) is particularly suitable owing to its biocompatibility, low cost, commercial availability and favorable adsorption behaviour on metal-oxide surfaces [23]. PEG adsorbs onto ZnO principally through hydrogen bonding involving the surface hydroxyl groups of the oxide [24], with a complementary, backbone-distributed contribution from coordinative donation of the ether oxygen lone pairs to Lewis-acidic Zn^2+^ surface sites [25]. PEG encapsulation has been shown to limit ZnO crystal growth and improve monodispersity [26,27,28], and PEG-coated ZnO has been incorporated successfully into an aliphatic polyester matrix [29]. In the present system, the adsorbed layer is expected to act through steric stabilization, through reduction in surface energy by screening the polar surface hydroxyls, and through improved interfacial compatibility, since the PEG ether backbone can engage in dipole–dipole interactions and weak hydrogen bonding with the ester linkages of both PLA and PBS.

To the best of our knowledge, surface-modified ZnO combined with carbon fiber in a biodegradable PLA/PBS matrix has not previously been reported for EMI shielding. Here, ZnO nanoparticles were coated with PEG (8 kDa) by solution adsorption and incorporated at finely graded loadings of 0.5, 1, 1.5 and 2 wt % into PLA/PBS (80/20) containing a fixed 20 wt % CF, using a processing protocol identical to that established for the unmodified-ZnO system so that the surface treatment is the only deliberate variable. Surface anchoring was verified spectroscopically, and dispersion, mechanical, thermal and X-band shielding behaviour were then characterized across the composition series and compared against the unmodified-ZnO baseline. Each property is assessed against the requirements set out above rather than in isolation, so that the composition recommended for use is the one satisfying all of them simultaneously rather than the one giving the highest shielding effectiveness. The agglomeration-driven embrittlement is eliminated, and absorption-dominant shielding of 89 dB is reached at only 1.5 wt % nanoparticle content. The composition delivering the best mechanical performance is not the one delivering the highest shielding, so the dispersion–mechanics–shielding trade-off can be navigated deliberately rather than encountered as a limitation.

## 2. Materials and Methods

### 2.1. Materials

The materials used in this study are listed in Table 1. All reagents were used as received.

### 2.2. PEG Surface Modification of ZnO Nanoparticles

PEG-coated ZnO (denoted PZ) was prepared by solution adsorption in ethanol. ZnO nanoparticles (1.0 g) were dispersed in 80 mL of ethanol (≈1.25 wt % solids) and probe-ultrasonicated at 40% amplitude for 45 min in an ice-water bath to break up loose aggregates. The ice bath suppresses localized heating during sonication. PEG (0.1 g, corresponding to 10 wt % relative to ZnO) was dissolved separately in 20 mL of ethanol by magnetic stirring for 30 min and added dropwise to the ZnO suspension under continuous stirring at 400 rpm. The suspension was then stirred at 400 rpm and 45 °C for 6 h to allow the adsorbed layer to equilibrate. This temperature lies well below the melting point of PEG 8 kDa (≈60–65 °C). Bulk ethanol was removed by rotary evaporation at 45 °C under reduced pressure, and the recovered powder was vacuum-dried at 45 °C for 24 h. The PEG loading (10 wt % relative to ZnO) and the 8 kDa molecular weight were selected to provide a steric layer thick enough to stabilize dispersion while keeping the insulating organic fraction low, since interfacial polarization and conductive contact within the CF network are sensitive to interlayer thickness [24].

### 2.3. Composite Fabrication

The matrix ratio (PLA/PBS = 80/20 wt %) and the CF content (20 wt %) were held constant while the PZ content was varied. The compositions are summarized in Table 2.

PLA and PBS granules were vacuum-dried at 80 °C for 12 h to remove residual moisture and prevent hydrolytic degradation during extrusion. PLA, PBS and PZ were fed simultaneously into a co-rotating twin-screw micro-compounder (MC 15 HT, Xplore Instruments, Sittard, The Netherlands) and melt-blended at a barrel temperature of 180 °C and a screw speed of 60 rpm with a residence time of 2.15 min. Once a homogeneous melt had been achieved, CF was introduced and blended for a further 45 s to ensure dispersion while minimizing fiber breakage. Extrudates were injection-molded (MI 12, Xplore Instruments) at a melt temperature of 180 °C, a mold temperature of 25 °C and an injection pressure of 10 bar, producing tensile specimens and rectangular sheets of 4 × 8 × 0.2 cm^3^ for EMI characterization. Nitrogen purging was maintained throughout extrusion to suppress thermo-oxidative degradation. This protocol reproduces that used for the unmodified-ZnO system [14], so that the PEG coating is the only deliberate variable between the two data sets.

### 2.4. Characterization

#### 2.4.1. Structural Characterization

Structural characterization was performed by Fourier-transform infrared spectroscopy (FTIR; PerkinElmer, Spectrum 100, Waltham, MA, USA) over 650–4000 cm^−1^ at a resolution of 4 cm^−1^ with 8 co-added scans.

#### 2.4.2. Tensile Test

Tensile properties were determined on dog bone specimens according to ISO 527-2:2012 (Geneva, Switzerland) [30] (overall length 50 mm, gauge length 25 mm, width of the narrow Section 4 mm, thickness 2 mm) at a crosshead speed of 5 mm min^−1^, with four specimens tested per composition. The protocol was identical to that used for the reference materials [14] so that the two data sets are directly comparable. Individual stress–strain curves are provided in Figure S3 and averaged values, reported with the standard error, in Table 3. Differences between compositions were assessed with Welch’s two-sided *t*-test (*n* = 4 per composition) at a significance level of 0.05.

#### 2.4.3. Differential Scanning Calorimetry

Thermal transitions were measured by differential scanning calorimetry (DSC; Mettler Toledo, DSC1, Greifensee, Switzerland) under nitrogen. The program consisted of two heating cycles from −50 to 200 °C and one intermediate cooling cycle from 200 to −50 °C, all at 10 °C min^−1^. Glass transition, melting and cold crystallization temperatures (T_g_, T_m_, T_cc_) and the corresponding enthalpies were taken from the second heating run.

Conventionally, the degree of crystallinity (X_c_) of a polymer is calculated by subtracting the polymer cold-crystallization enthalpy from its melting enthalpy. In the present PLA/PBS system, however, the apparent cold-crystallization event overlaps with PBS phase related thermal transitions. Consequently, an apparent melting enthalpy-based PLA crystallinity was calculated for comparative purposes as

X_c_ = [ΔH_m_/(ΔH_m_° × w)] × 100 (1)

where ΔH_m_ is the measured melting enthalpy, ΔH_m_° is the melting enthalpy of 100% crystalline PLA (93.0 J g^−1^) [31] and w is the weight fraction of the polymer.

#### 2.4.4. Thermogravimetric Analysis

Thermal stability was assessed by thermogravimetric analysis (TGA; Mettler Toledo, TGA 1, Greifensee, Switzerland) from 25 to 600 °C at 10 °C min^−1^ under nitrogen, from which the degradation temperatures at 5 and 50 wt % mass loss (T_d5_, T_d50_), the maximum-rate temperatures (T_max_) and the residual mass at 600 °C were extracted.

#### 2.4.5. Morphology

Fracture-surface morphology was examined by scanning electron microscopy (Brand: Thermo Scientific, Model: Quattro S SEM, Waltham, MA, USA) on cryogenically fractured, gold-coated specimens. The ZnO and PZ powders were additionally imaged in bright-field scanning transmission electron microscopy (STEM) (Brand: Thermo Scientific, Model: Quattro S SEM, Waltham, MA, USA) mode at 30 kV to establish particle size and habit. The adsorbed organic layer lies at or below the resolution limit of this configuration and was not measured from these images [32].

#### 2.4.6. EMI Measurement

EMI shielding performance was measured over the X-band (8.2–12.4 GHz) using a two-port vector network analyser (Rohde & Schwarz, ZVB20, Munich, Germany) equipped with a WR-90 waveguide, following a full two-port TOSM (through–open–short–match) calibration at the coaxial cable ends. Measurements were made on injection-molded plates of 80 × 40 × 2 mm, which cover the full 22.86 × 10.16 mm aperture of the WR-90 flanges. All EMI shielding measurements were performed using three independently prepared specimens for each composition to confirm the reproducibility of the results. The reported shielding effectiveness values represent the average response obtained from the replicate measurements.

### 2.5. EMI Shielding Theory and Parameter Extraction

The scattering parameters S_11_ and S_21_ were measured, from which the reflection (R), transmission (T) and absorption (A) power coefficients were obtained as R = |S_11_|^2^, T = |S_21_|^2^ and A = 1 − R − T. The shielding contributions were then calculated as

SE_R_ = −10 log_10_(1 − R) (2)

SE_A_ = −10 log_10_[T/(1 − R)] (3)

SE_T_ = SE_R_ + SE_A_ + SE_M_ (4)

A_eff_ (%) = [A/(1 − R)] × 100 (5)

where SE_M_, the contribution of multiple internal reflection, is neglected for SE_T_ > 10 dB. The dominant attenuation mechanism was identified from the SE_A_/SE_T_ ratio, absorption dominance being indicated by SE_A_ > SE_R_ together with SE_A_/SE_T_ > 0.8.

## 3. Results and Discussion

### 3.1. Structural Characterization by FTIR

#### 3.1.1. Verification of PEG Anchoring on ZnO

**Figure 1:**
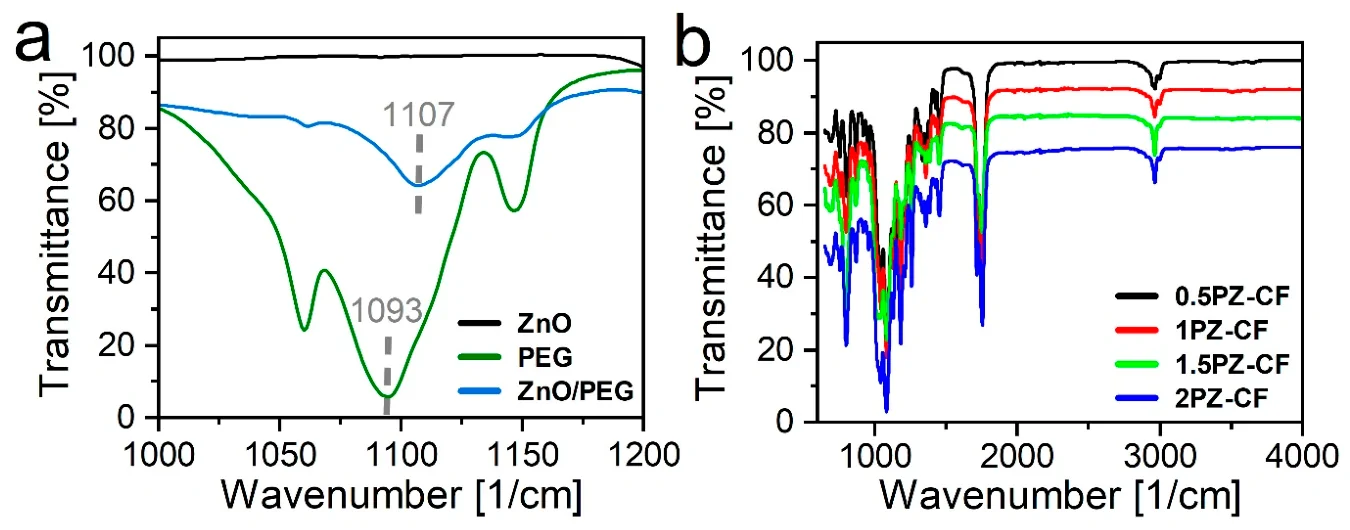
(**a**) FTIR spectra of neat ZnO, neat PEG and PEG-coated ZnO in the C–O–C stretching region, showing the shift in the dominant ether component from 1093 to 1107 cm^−1^ upon adsorption; (**b**) full-range FTIR spectra of the PLA/PBS/CF composites containing 0.5–2 wt % PEG-coated ZnO.

Figure 1a compares the FTIR spectra of as-received ZnO, neat PEG (8 kDa) and the PEG-coated powder over the 1000–1200 cm^−1^ interval, which contains the ether C–O–C stretching absorption. This is the strongest and most diagnostically informative band of PEG. Uncoated ZnO measured under identical conditions stays between [98 and 100]% transmittance across the interval, indicating that any absorption observed in the coated powder originates unambiguously from the adsorbed polymer, and no spectral subtraction is required.

Neat PEG exhibits the multi-component C–O–C envelope characteristic of a semicrystalline polyether, with resolved contributions near 1060 and 1145 cm^−1^ flanking a dominant component at 1093 cm^−1^. In the coated powder, the envelope is markedly altered. The dominant component now appears at 1107 cm^−1^, a blue shift of +14 cm^−1^, and the resolved side components are largely lost in favour of a single broadened band.

A displacement of this magnitude cannot arise in a physical mixture, in which the PEG chains would remain in a bulk environment, and the band would be unshifted. The blue shift therefore suggests that the ether oxygens along the PEG backbone are directly engaged with the ZnO surface.

#### 3.1.2. Origin of the Blue Shift

In the bulk semicrystalline state, PEG adopts a 7/2 helical conformation in which the O–CH_2_–CH_2_–O sequences are predominantly gauche about the C–C bond, and this conformation fixes the ether stretching frequency of the neat polymer [33]. Chains adsorbed flat against an oxide surface are conformationally constrained due to extension towards a trans-rich, more planar geometry which allows a larger number of ether oxygens to contact the surface simultaneously. Therefore, the associated stiffening of the backbone raises the effective force constant of the C–O–C oscillator, displacing the band to higher wavenumber. Conformational sensitivity of this kind is well established for ethylene glycol layers confined at solid surfaces, where the relative populations of helical and extended states are governed by substrate chemistry and packing density, and where a shift in the ether envelope to higher wavenumbers accompanies growth of the trans population [34,35].

The concurrent loss of the resolved side components provides a second, complementary line of evidence. The C–O–C absorption of semicrystalline PEG is a composite envelope whose components reflect distinct crystalline and amorphous chain populations, and this structure collapses towards a single band when the polymer is rendered amorphous [36]. A layer adsorbed on a nanoparticle surface is too strongly confined to crystallize as bulk PEG does, so the observed simplification of the envelope is exactly what surface confinement predicts. The PEG in the coated powder is therefore in an adsorbed, surface-constrained state rather than a bulk one.

The underlying interfacial chemistry is dual. PEG binds to ZnO principally through hydrogen bonding involving the surface hydroxyl groups of the oxide [24], while coordinative donation from the ether oxygen lone pairs to Lewis-acidic Zn^2+^ sites provides a second, backbone-distributed anchoring pathway [25]. The latter is consistent with the fact that the spectroscopic perturbation registers on the backbone ether mode, rather than on the chain-end hydroxyls alone. Multi-point anchoring of this type generates the steric layer exploited in the remainder of this work, with loops and tails extending into the surrounding medium to oppose interparticle approaches while screening the polar surface that otherwise drives aggregation.

#### 3.1.3. FTIR of the PLA/PBS/CF/PZ Composites

Figure 1b shows the full-range spectra of the four PZ-containing composites. All display the characteristic absorptions of the polyester matrix: the ester carbonyl stretching of PLA at approximately 1748 cm^−1^, C–O–C stretching vibrations at approximately 1180, 1082 and 1042 cm^−1^, CH_3_ and CH_2_ deformation bands between 1360 and 1452 cm^−1^, and C–H stretching absorptions in the 2850–3000 cm^−1^ region. Across the entire series these positions are unchanged and no new bands appear. The carbonyl envelope in particular shows neither displacement nor asymmetric broadening at any PZ loading, which is the signature expected if the ester groups of the matrix engaged in specific chemical interactions with the filler. The interactions among the matrix, the carbon fibers, and PZ are therefore predominantly physical, and the chemical constitution of the matrix is preserved through compounding and injection molding. The PEG treatment introduces no reactive interfacial chemistry, instead, its role is interfacial and morphological rather than chemical.

The PEG features resolved in Figure 1a are not detectable in the composite spectra, which is expected based on quantitative grounds rather than being evidence of coating loss during melt processing. PEG is present at 10 wt % relative to ZnO, so at PZ loadings of 0.5–2 wt % the PEG content of the composite is only 0.045–0.18 wt %, corresponding to 0.06–0.23 wt % relative to the polymer phase. The ether stretch moreover falls directly beneath the intense C–O–C envelope of the polyester at 1180–1042 cm^−1^ and cannot be separated from it. Therefore, FTIR of the bulk composite is not a sensitive probe of the coating. Verification of the coating rests on the powder-level spectra of Figure 1a.

### 3.2. Dispersion of PEG-Coated ZnO Nanoparticles

#### 3.2.1. Particle-Level Morphology of the Coated Powder

Bright-field STEM images of the as-received and PEG-coated powders are compared in Figure S1. Both samples consist of faceted, equiaxed ZnO particles. The structures resolved at this magnification are considerably larger than the nominal 18 nm primary size quoted by the supplier, indicating that the as-received powder is supplied as sintered polycrystalline particles rather than as discrete primary crystallites. Bright-field STEM at 30 kV resolves particle habit but does not resolve the adsorbed layer.

The functional significance of the adsorbed layer is that it imposes a finite minimum separation between approaching particles and thereby opposes the van der Waals attraction that otherwise drives loose assemblies to densify into hard agglomerates [24,29]. Loose assemblies held together by weak interactions are dispersible under the shear field of a twin-screw compounder, whereas hard agglomerates survive melt compounding intact and persist in the final composite as defects.

#### 3.2.2. Composite Fracture Surfaces

**Figure 2:**
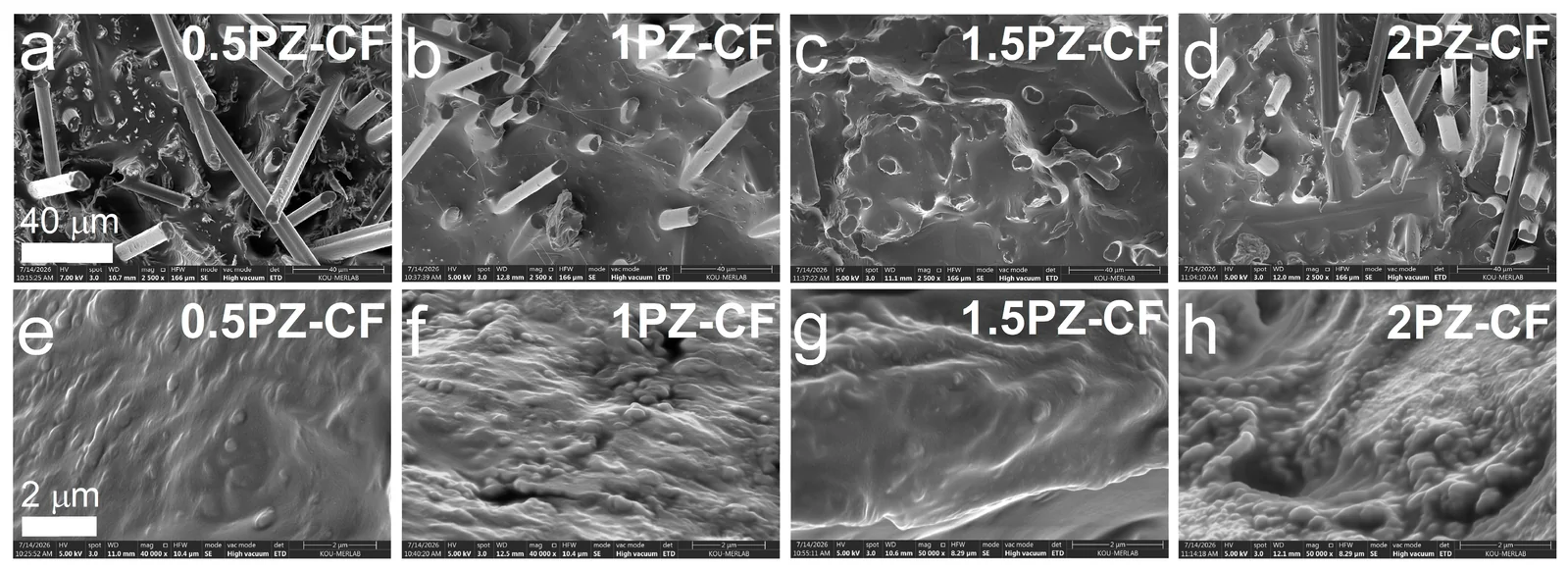
SEM micrographs of the fracture surfaces of PLA/PBS/CF composites containing 0.5, 1, 1.5 and 2 wt % PEG-coated ZnO: (**a**–**d**) ×2500, scale bar 40 µm; (**e**–**h**) ×40,000–50,000, scale bar 2 µm.

Figure 2 shows fracture surfaces of the PZ composites at two magnifications. At ×2500 (Figure 2a–d), all four compositions exhibit a predominantly fibrous morphology, with fracture occurring along the fiber–matrix interface. The elongated structures observed in these micrographs correspond to carbon fibers. No systematic differences in fiber geometry, spatial distribution or fiber-specific surface features are observed across the series. Since the CF content was fixed at 20 wt % and the compounding sequence was identical for all samples, this invariance confirms that the incorporation of PZ does not modify the carbon fiber architecture, and that property differences across the series originate in the nanoparticle phase rather than in changes to the fiber network.

At ×40,000–50,000 (Figure 2e–h), discrete nanoparticulate features are resolved on the fracture surfaces, distributed across the matrix and in the regions surrounding the fibers. In all four compositions these features are sub-micron in scale, and no micron-scale secondary phase or compact particle cluster is detected, up to and including the highest loading of 2 wt %. Their size range is consistent with the particles imaged by STEM in Figure S1, indicating that the coated particles were carried through compounding without densifying into agglomerates.

#### 3.2.3. Direct Comparison with Unmodified ZnO

The effect of the surface treatment is isolated in Figure S2 is SI, which compares composites of otherwise identical composition and processing history containing 1 wt % PEG-coated ZnO and 1 wt % unmodified ZnO. In the composite containing unmodified ZnO, the nanoparticles exhibit a highly non-uniform distribution, with pronounced accumulation in dense, contiguous bands along the fiber–matrix interface. In contrast, the adjacent matrix regions are comparatively depleted of nanoparticles. In the composite containing coated particles, no such accumulation occurs and the nanoparticulate phase is distributed evenly across the observed area at sub-micron scale.

Because the two samples differ only in the surface state of the nanoparticles, this comparison attributes the improvement in dispersion directly to the PEG layer, and not to the loading level, the compounding conditions or the presence of the carbon fibers. The mechanism is consistent with the interfacial chemistry suggested in Section 3.1. The adsorbed layer provides a steric barrier that opposes the approach of neighbouring particles and simultaneously screens the polar surface hydroxyls responsible for the high surface energy of bare ZnO. This reduces the thermodynamic driving force for aggregation and enhances compatibility with the hydrophobic polyester matrix [24,29,37]. Comparable improvements have been reported for ZnO subjected to other surface treatments in PLA-based systems, including silanization [38] and polydopamine functionalization [39], indicating that the effect is general to surface passivation rather than specific to PEG.

The morphological evidence presented here therefore provides the structural basis for the mechanical and shielding behaviour reported in the following sections. Nevertheless, it should be emphasized that electron microscopy examines only a limited area of the fracture surface, making these observations inherently local. The implications of these microstructural features at the bulk scale are therefore assessed through the mechanical and electromagnetic measurements presented below.

### 3.3. Tensile Properties

#### 3.3.1. Deformation Behaviour

The individual stress–strain curves of the four PZ composites are presented in Figure S3, with four specimens per composition, and the averaged parameters with the standard error are summarized in Table 3. The replicate curves overlay closely and reveal a characteristic two-stage response. An initial linear region terminates in a small discontinuity at approximately 0.35% strain, visible as a brief load drop followed by recovery. This event is attributed to the onset of localized fiber–matrix debonding, consistent with its appearance at a comparable strain in every specimen and every composition. The material then continues to carry increasing load through an extended region up to a maximum, before failing.

This behaviour differs from that of the unmodified-ZnO system, in which composites containing 3, 5 and 7 wt % ZnO failed at strains of 0.30, 0.18 and 0.06% respectively [14]. In the unmodified system the composite therefore fractures at the first damage event, whereas the PZ composites survive it and continue to carry load to strains five to ten times greater. Because the tabulated stress and strain correspond to the maximum of this two-stage curve, the values in Table 3 are reported as strength and strain at maximum force.

#### 3.3.2. Effect of Carbon Fiber and PZ Content

Incorporation of 20 wt % CF transforms the mechanical response of the matrix: tensile strength rises from 23.66 ± 2.11 to 70.75 ± 3.79 MPa and the modulus from 4252 ± 971 to 17,203 ± 2784 MPa, confirming efficient load transfer into the fiber network. This reinforcement is obtained at a severe cost in ductility with the tensile strain falling from 9.33 ± 1.03% to 1.31 ± 0.11%. Such strength–ductility trade-offs are common in polyesters. In uniaxially drawn terpolyester films, for example, tensile strength increased with molecular orientation while the strain at break decreased [40]. The PLA/PBS/CF composite therefore defines the reference state against which the nanoparticle phase must be assessed: any filler added to this system must improve, or at minimum not further degrade, an already embrittled material.

All four PZ composites retain substantially greater ductility than this reference. Tensile strain decreases monotonically with PZ loading, from 3.09 ± 0.23% at 0.5 wt % through 2.35 ± 0.17 and 2.19 ± 0.24% to 1.92 ± 0.17% at 2 wt %, but even the highest loading remains 47% above the CF reference. The increase in strain relative to PLA/PBS/CF is statistically significant for every PZ composition (+47 to +136%; *p* ≤ 0.03, Welch’s *t*-test, *n* = 4). The strength response is non-monotonic and has its highest mean value at 1 wt %: it rises from 59.35 ± 6.77 MPa at 0.5 wt % to 76.30 ± 1.75 MPa at 1 wt %, then declines through 73.16 ± 3.16 MPa to 65.09 ± 2.61 MPa at 2 wt %. None of these values differs significantly from the strength of PLA/PBS/CF (*p* = 0.21–0.64); within the series, only the difference between 1 and 2 wt % is significant (*p* = 0.015). The 1PZ-CF composition is notable in that its tensile strength equals that of the PLA/PBS/CF reference within experimental uncertainty (76.30 ± 1.75 against 70.75 ± 3.79 MPa), while its strain is 79% higher. Gains in ductility without a loss of strength are uncommon in particulate-filled systems, where the two are normally traded against one another, and are attainable only when the nanofiller is both well dispersed and well bonded to the surrounding matrix [41,42]. The decline in strength from 1 to 2 wt %, together with the progressive decrease in strain across the series (*p* = 0.008 between 0.5 and 2 wt %), indicates that the benefit saturates and then reverses, consistent with residual particle–particle contact becoming increasingly probable as the interparticle spacing decreases.

The modulus behaves differently from strength and ductility, with all PZ compositions falling below the CF reference by 13% at 1 wt % and by approximately 22% at 1.5 and 2 wt %. These differences are not statistically significant (*p* = 0.28–0.51), owing to the large specimen-to-specimen scatter of the reference modulus, whose standard error is 16% of the mean. The 1PZ-CF composition therefore retains both the strength and the modulus of the reference within experimental uncertainty. The consistently lower mean modulus of the PZ series is nevertheless examined in Section 3.3.4. The 0.5PZ-CF composition departs from the trend of the series, with a modulus of 8549 ± 1371 MPa, the only value significantly below that of the reference (*p* = 0.045). The individual curves in Figure S3a confirm that the lower initial stiffness is consistently observed across all four specimens and is therefore unlikely to be a single-specimen artefact. However, because no monotonic relationship with filler content is evident, this parameter should not be assigned mechanistic significance without further experimental replication.

#### 3.3.3. Comparison with Unmodified ZnO

The surface treatment is assessed by comparison with the unmodified-ZnO composites of identical matrix and fiber composition [14]. Because the PLA/PBS and PLA/PBS/CF reference values are common to both data sets, the comparison is internally consistent. At a matched loading of 1 wt %, surface modification raises the tensile strain from 1.66 to 2.35% (+42%), the strength from 55.17 to 76.30 MPa (+38%) and the modulus from 13,274 to 14,939 MPa (+13%), so that all three properties improve simultaneously.

The contrast becomes more pronounced across the composition series. In the unmodified system, tensile strain collapses beyond 1 wt %, reaching 0.30% at 3 wt %, 0.18% at 5 wt % and 0.06% at 7 wt %, while strength declines monotonically to 28.53 MPa; no unmodified-ZnO composition exceeds the strength of the CF reference. In the PZ system, by contrast, tensile strain never falls below 1.92%, strength remains between 59 and 76 MPa across the whole range, and two compositions exceed the CF reference. The severe embrittlement observed in the unmodified system is therefore absent. This behavior is consistent with the morphological evidence presented in Section 3.2, where the interfacial particle-rich bands associated with premature fracture in the unmodified composites are no longer observed after ZnO coating.

#### 3.3.4. Mechanistic Interpretation

Two mechanisms have been considered to account for the tensile improvement: enhanced dispersion and plasticization of the matrix by PEG. The data support the first and limit the second to a localized form.

The dispersion contribution is well supported. The coating prevents the dense interfacial ZnO bands formed by unmodified ZnO at the same loading (Figure S2). Agglomerates act as stress concentrators and crack initiators and reduce the effective particle–matrix interphase [41,42]. Elimination of particle accumulations removes the dominant defect population, restores the nanoparticle–matrix interfacial area, and preserves the continuity of stress transfer through the fiber network. This is the necessary condition for the increase in strain obtained without a loss of strength at 1 wt %, and it explains why the improvement degrades progressively with increasing loading as the interparticle spacing decreases.

Bulk plasticization, by contrast, is not consistent with the data. First, the quantity of PEG present is far too small: at 0.045–0.18 wt % of the composite, it is between one and two orders of magnitude below the 5–20 wt % typically required for PEG to plasticize PLA effectively [43]. Second, plasticization necessarily manifests as a depression of T_g_, and the DSC results in Section 3.4 show none: T_g_ of the PLA phase remains within 56.0–57.8 °C across the PZ series against 57.2 °C for the CF reference, with no monotonic dependence on PZ content. Third, the dose–response is inverted relative to the plasticization prediction; a plasticizer would increase elongation and decrease modulus progressively with increasing content, whereas the measured tensile strain decreases monotonically with PZ loading while the modulus is essentially constant from 1 to 2 wt %. Fourth, the FTIR results establish that the PEG is anchored to the ZnO surface rather than dispersed as free polymer, so it is not available to increase bulk chain mobility.

A localized form of the second mechanism is consistent with the data. The adsorbed PEG constitutes a thin, compliant organic interlayer confined to the immediate vicinity of each nanoparticle. Soft interphases of this kind are known to enhance toughness in particulate composites by permitting local shear deformation and by dissipating the energy of an advancing crack front at the particle boundary, without altering the bulk glass transition of the matrix [41]. This interpretation is consistent with the overall pattern of results. The increased ductility can be attributed to local energy dissipation within the compliant interphase, while the retained strength reflects the preservation of effective interfacial load transfer. The same interphase can also account for a lower mean modulus alongside a retained strength, since the modulus is governed by the initial linear response and the maximum stress by the later stages of deformation, after fiber–matrix debonding has begun (Figure S3). At 0.1–0.4 vol %, the particles are too few for their own stiffness, or that of their coating, to lower the composite modulus appreciably, so the effect is more plausibly interfacial. The modulus of a short-fiber composite depends on the efficiency of shear stress transfer to the fibers, which decreases when particles at the fiber–matrix interface (Figure 2e–h) make it more compliant or allow limited slip. After debonding, the same layer delays the coalescence of damage, so that the maximum stress is retained [42,44]. The interphase interpretation is also consistent with the mild reduction in cold crystallization temperature observed at 0.5 and 1 wt % PZ (Section 3.4), which indicates locally enhanced chain mobility at the filler interface rather than throughout the matrix. On this basis, 1 wt % PZ represents the optimum composition for mechanical performance. Interfacial properties were not measured directly, and this interpretation rests on the tensile, morphological and thermal results.

### 3.4. Thermal Behaviour

#### 3.4.1. Thermal Transitions (DSC)

Thermal transition temperatures and the calculated crystallinity values obtained from the second heating curves are tabulated in Table 4.

In our earlier study, neat PLA exhibited a glass transition at 59.9 °C, a cold crystallization peak at 120.9 °C and a melting transition at 159 °C, without crystallization during the cooling cycle. Neat PBS does not exhibit a glass transition in this window; according to the manufacturer, its T_g_ lies near −35 °C. For neat PBS, a cold crystallization peak at 99.2 °C, a melting peak at 115 °C and a crystallization peak on cooling at 73 °C were detected, indicating a higher crystallization ability than PLA [13]. After blending, a single distinct T_g_ was observed for the PLA/PBS blend and for all composites in the range 55.3–57.8 °C, attributed to the PLA-rich phase. The decrease of approximately 5 °C relative to neat PLA is attributed to PBS slightly increasing the chain mobility of PLA and to the partial compatibility of the two polymers [45]. The addition of 20 wt % CF raised T_g_ from 55.3 to 57.2 °C, suggesting a modest restriction of PLA segmental mobility. Regarding the melting behaviour, the PLA/PBS blend exhibited two distinct endothermic transitions at 118.0 and 158.5 °C, attributed to the melting of the PBS and PLA phases, respectively. Since both polymers are semi-crystalline, both phases retained their characteristic crystalline melting behaviour after blending. The CF incorporation shifted these T_m_ values slightly to lower temperatures, namely, 156.8 °C for PLA and 115.6 °C for PBS, suggesting moderate changes in polymer chain packing and lamellar organization. On the other hand, CF affected melting enthalpies of polymers differently and ΔH_m−PLA_ raised from 20.3 to 23.1 J/g, whereas the apparent ΔH_m−PBS_ decreased dramatically from 16.6 to 0.8 J/g. However, the melting transition of neat PBS in blend must be interpreted with care, as the melting of neat PBS and the cold crystallization transition of neat PLA occur at close temperatures and overlap. As can be seen in table, the PLA/PBS blend exhibited a single apparent T_cc_ peak, which may contain overlapping contributions from PLA cold crystallization and PBS-related crystallization and melting; as also reported by Qahtani et al. [31] and Ostrowska et al. [46]. Therefore, the PBS melting enthalpy should be interpreted not as an independent measure of PBS crystal content, but rather as a net apparent endothermic contribution.

After the incorporation of PZ, the T_g_ values remained within a narrow interval of 56.0–57.8 °C without a monotonic dependence on content, demonstrating that PZ did not substantially alter the segmental mobility of the PLA phase. The T_g_ reduction was considerably more pronounced for unmodified ZnO-reinforced PLA/PBS/CF composites [14], and a similar contrast was observed for the PLA melting temperature. These findings are consistent with previous reports that ZnO accelerates PLA chain scission during extrusion, leading to molecular weight reduction and to degradation products that plasticize the matrix [47,48,49]. In the surface-coated case, T_m−PLA_ varied only slightly between 153.7 and 155.1 °C, whereas ΔH_m−PLA_ progressively decreased from 22.4 to 19.7 J/g with increasing PZ content. This suggests that PZ had a limited effect on the PLA melting temperature but gradually reduced its melting enthalpy. Although PZ particles restrict the PLA chain packing and crystal growth to some extent, this effect is less pronounced compared to the uncoated ZnO. The PEG layer partially screens the active ZnO surface and reduces direct ZnO–PLA contact, so that the weaker T_g_ and T_m_ depression observed in the PLA/PBS/CF/PZ composites aligns with the findings of Cunha et al. [47]. The melting behaviour of PBS phase showed a non-monotonic response to PZ incorporation and content. A melting temperature ranging from 109.2 to 114.4 °C was detected for the PZ composites, representing a slight reduction compared to the T_m−PBS_ of the PLA/PBS/CF composite. The apparent ΔH_m−PBS_ generally decreased from 10.1 J/g at 0.5PZ-CF to 2.9 J/g at 2PZ-CF. Considering the aforementioned overlap, this trend most likely reflects changes in the relative contributions of the PBS melting and the overlapping cold-crystallization event rather than a direct change in PBS crystal content.

While both neat PLA and neat PBS exhibit a cold crystallization peak, a single such peak was observed in the blends and composites, in the range 104–109 °C. As previously emphasized, in PLA/PBS systems, the cold crystallization of PLA may overlap with PBS-related crystallization or melting transitions [31,46,50]. The peak observed here was therefore treated as an apparent cold crystallization event rather than being attributed solely to PLA or PBS. The T_cc_ value of the PLA/PBS blend was 107.8 °C, and CF incorporation increased it slightly to 109.0 °C, indicating a modest restriction of chain rearrangement. PZ incorporation shifted T_cc_ to lower temperatures, particularly for 0.5PZ-CF and 1PZ-CF at 104.6 and 104.0 °C, indicating that PZ facilitates the onset of cold crystallization during heating, most likely by promoting earlier chain packing of the PLA phase. However, because the enthalpy of this apparent event could not be assigned uniquely to PLA owing to its overlap with PBS phase related transitions, ΔH_cc−PLA_ could not be determined reliably. Accordingly, the X_c−PLA_ values were calculated without considering the cold crystallization enthalpy. A similar approach has also been applied in previous studies [31,46,50]. The X_c−PLA_ values calculated without considering the cold crystallization enthalpy revealed only a slight decrease in X_c−PLA_ decreased gradually from 38.8% for PLA/PBS/CF to 33.9% for 2PZ-CF, indicating that the earlier onset of cold crystallization did not result in a higher PLA crystalline fraction.

#### 3.4.2. Thermal Stability (TGA)

**Figure 3:**
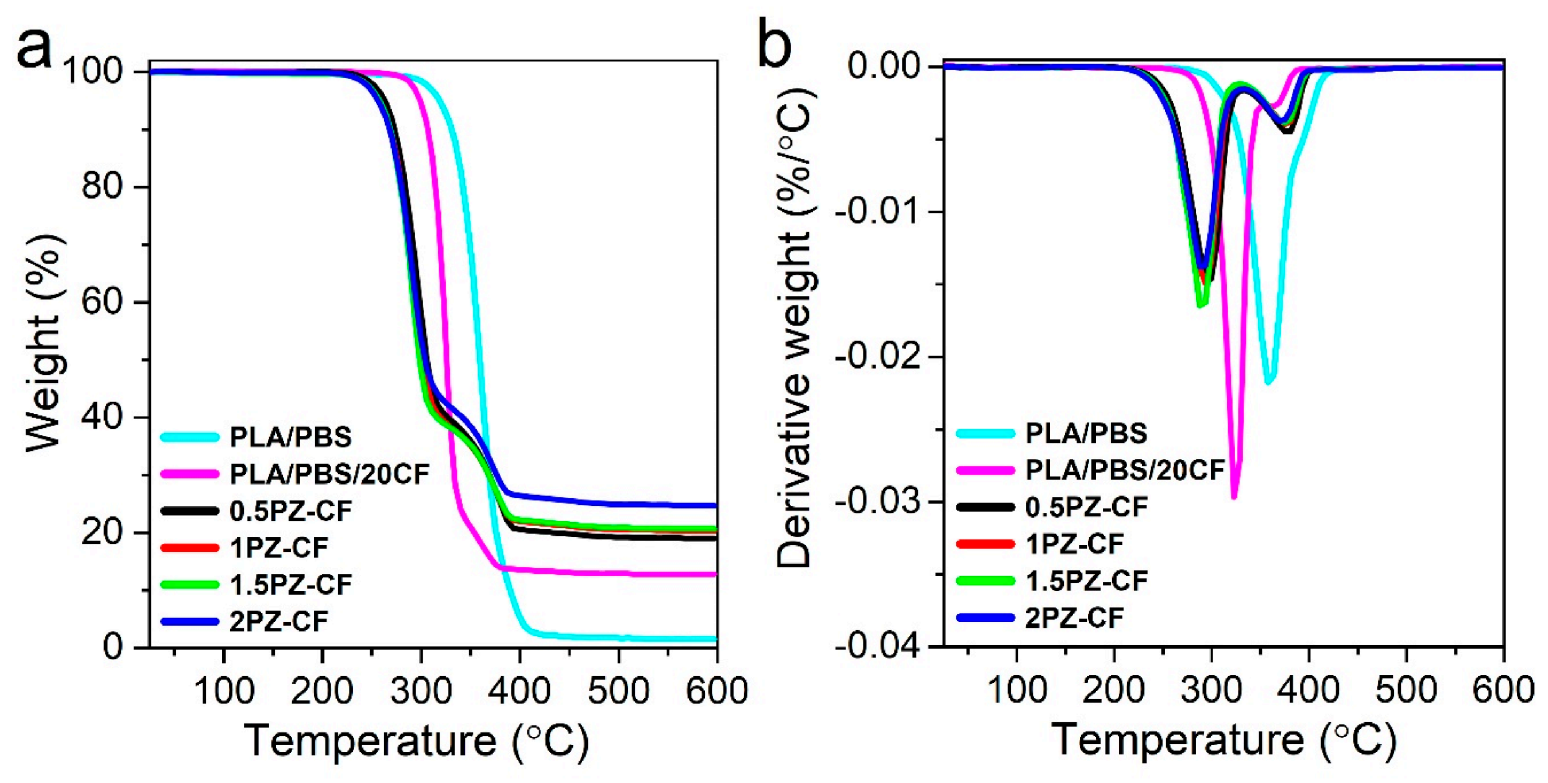
(**a**) TGA and (**b**) DTG curves of the PLA/PBS blend, the PLA/PBS/CF composite and the PZ-containing composites.

Thermal stability is a complementary design parameter for EMI shielding materials. Under service conditions, sustained or intermittent thermal exposure may cause matrix degradation, compromising both the structural integrity of the composite and the continuity of the filler network that contributes to shielding. The TGA and derivative (DTG) curves are presented in Figure 3, and the corresponding degradation temperatures and residual weights are given in Table 5.

As reported in our previous study [13], neat PLA and PBS exhibit single-step degradation curves with maximum degradation peak temperatures at 363 and 395 °C, respectively. The PLA/PBS blend showed degradation temperatures between those of the two neat polymers, with an initial degradation point at 318.7 °C. As seen in Figure 3b, a single-step degradation curve was monitored at T_max_−1 of 359.8 °C with a small shoulder near 390 °C, corresponding to PLA and PBS decomposition, respectively. Considering the CF-reinforced composite, CF reinforcement shifted all characteristic degradation temperatures of the blend matrix to lower values, suggesting an accelerated matrix degradation response. The increased intensity of the primary DTG peak of the PLA/PBS/CF composite relative to the blend supports this interpretation, and the second degradation peak, observed only as a small shoulder in the blend, became more pronounced with the addition of CF. The T_max_−1 and T_max_−2 values decreased by 25 and 30 °C, respectively, upon CF inclusion. Similar reductions have been reported for melt-blended PLA/TPU/CF [51] and 3D-printed PLA/CF [52] composites. This adverse effect, despite the high intrinsic thermal stability of CF, indicates that its thermal influence is mediated by other factors including the polymeric matrix, processing history, interfacial interactions and contamination.

The addition and increasing content of PEG-coated ZnO nanoparticles also generally decreased the T_d5_, T_d50_ and T_max_−1 values. In particular, the marked decreases in T_d5_ and T_max_−1, assigned to main-chain degradation of PLA, demonstrate that the PZ nanofiller promotes early-stage degradation of the PLA-rich phase, consistent with the reported catalytic effect of ZnO on polyester degradation [14,21,48]. The interactions between active ZnO surface sites and the ester groups of PLA phase may facilitate chain scission during melt compounding, producing lower-molecular-weight chains that degrade at lower temperatures. In unmodified ZnO-filled PLA/PBS/CF composites, the same reduction trend was observed in T_d5_ and T_max_−1 while T_max_−2 remained almost constant. In contrast, T_max_−2 shifted from 364.7 °C for PLA/PBS/CF to 371.9–378.3 °C in the PZ-filled composites, and the intensity of the T_max_−2 peak attributed to PBS degradation also increased with PZ addition. PZ therefore induced a differentiated thermal response, comprising an accelerated low-temperature event and a delayed high-temperature event associated predominantly with the PBS-rich phase, together with an increased residual weight. Overall, compared with the previously reported unmodified ZnO-reinforced composites, composites containing PZ at the same nominal filler loading exhibited less weakening of thermal stability. This behaviour is attributed to partial screening of the ZnO surface by the PEG layer, which reduces direct ZnO–polyester contact, and is consistent both with the DSC findings above and with previous PLA/ZnO studies showing that ZnO surface passivation by lactic acid [47], polydopamine [39] or silanization [38] mitigates ZnO-induced PLA degradation. Improved nanoparticle dispersion may further delay thermal degradation by reducing the number of agglomerates that act as local thermal stress points [47].

### 3.5. Electromagnetic Interference Shielding Performance

#### 3.5.1. Shielding Effectiveness and Power Coefficients

**Figure 4:**
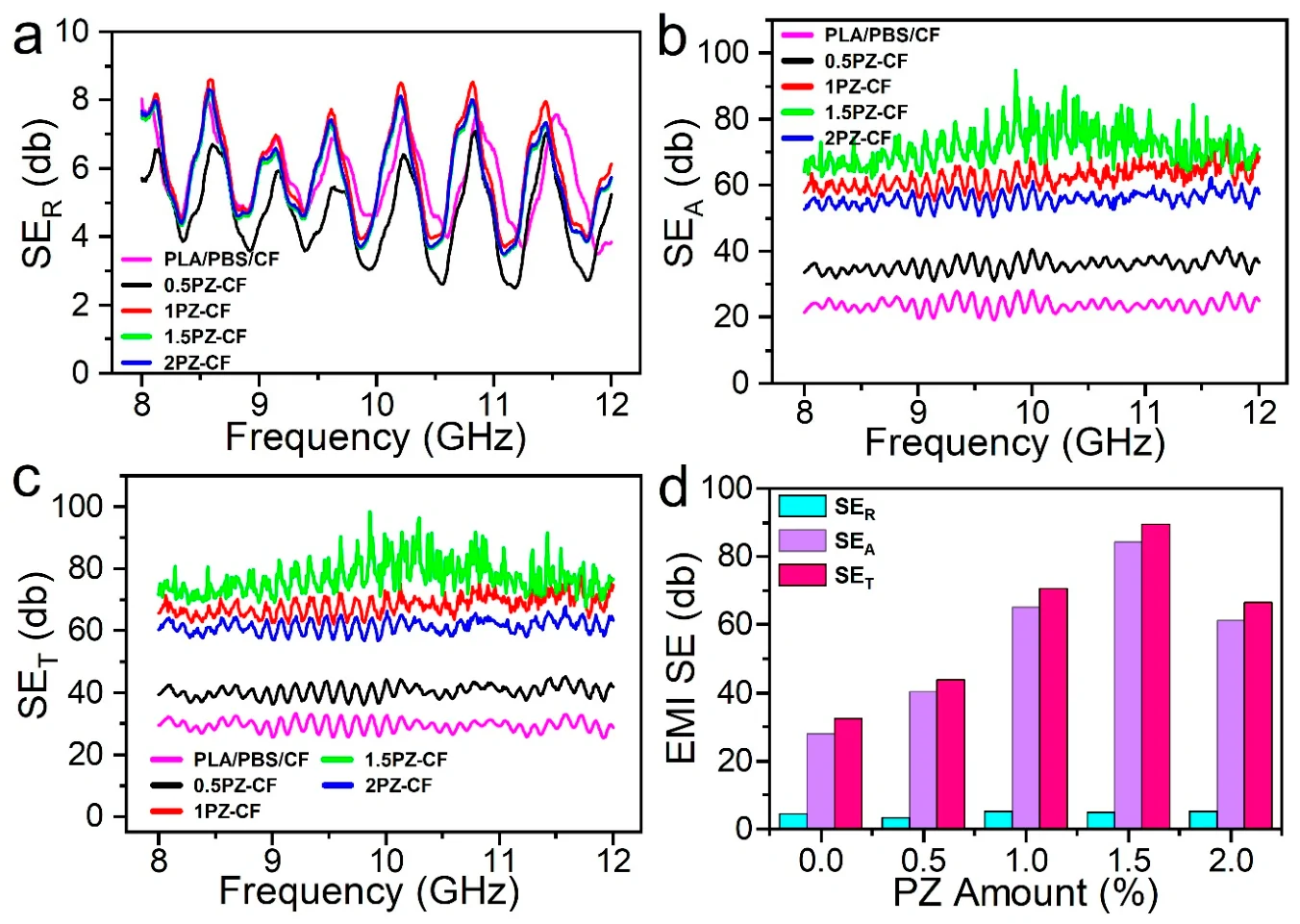
Variation of (**a**) SE_R_, (**b**) SE_A_ and (**c**) SE_T_ of the PLA/PBS/CF/PZ composites with frequency in the X-band, and (**d**) comparison of SE_R_, SE_A_ and SE_T_ as a function of PZ content at 10 GHz.

EMI shielding tests were carried out over the X-band (8–12 GHz) and evaluated in terms of the reflection (SE_R_), absorption (SE_A_) and total (SE_T_) shielding effectiveness. As shown in Figure 4a, all samples exhibit comparatively low SE_R_ values in the range of approximately 3–8 dB, with only a slight increase as the PZ content rises. This indicates that reflection is not the primary attenuation mechanism and that the composites present good impedance matching with free space. A high free-electron density alone is insufficient for effective shielding in polymer composites; impedance matching and the energy dissipation mechanisms operating within the internal structure of the material are equally decisive, and low SE_R_ values are characteristic of conductive carbon-reinforced composites in which absorption dominates [53,54,55].

The absorption-based shielding effectiveness in Figure 4b shows that the incorporation of PEG-coated ZnO markedly improves the ability of the composite to attenuate microwave energy. Whereas SE_A_ for the PLA/PBS/CF composite remains comparatively low, the addition of 0.5–1.5 wt % PZ produces a progressive and substantial improvement. This is attributed to the steric repulsion provided by the PEG shell, which lowers the van der Waals attraction between ZnO nanoparticles and prevents aggregation [37]. The resulting uniform distribution increases the number of ZnO–matrix and ZnO–fiber interfaces at which electromagnetic energy can be dissipated as heat [9,56,57]. The loss mechanisms through which these interfaces can contribute in the X-band are discussed in Section 3.5.2.

The total shielding effectiveness in Figure 4c follows a trend almost identical to that of SE_A_, confirming that absorption governs the overall attenuation. The PLA/PBS/CF composite exhibits SE_T_ of 30.7 dB at 10 GHz, whereas the composites containing 0.5, 1 and 1.5 wt % PZ reach 43, 71, and 89 dB, respectively. Values above 80 dB are generally regarded as ultra-high performance, 40 dB as high performance, and 20 dB as adequate for commercial purposes [6,7,8]. The composite with 1 wt % PZ already delivers 71 dB, more than three times the 20 dB commercial threshold and well above the 30–40 dB required for sensitive equipment.

The value of 89 dB at 1.5 wt % is a laboratory result for 2 mm plates and exceeds what enclosures for short-lifecycle electronics require. For these applications the relevant measure is the nanoparticle loading at which the requirement is met, because the loading also governs whether the mechanical and processing constraints can be satisfied. On this basis 1 wt % PZ is the preferred composition, because it gives 71 dB together with the highest tensile strength of the series. The remaining margin could be used to reduce wall thickness, since SE_A_ scales approximately with thickness [6]. This has not been tested here.

Figure 4d further shows that SE_A_ accounts for more than 90% of SE_T_ in every PZ composite, confirming absorption dominance across the entire composition range.

This enhanced performance arises from the combination of the conductive carbon-fiber network with the dispersed ZnO nanoparticles. The fibers establish conductive networks that promote conduction loss and multiple internal reflections, and the nanoparticles add polarization loss at sites connected to this network (Section 3.5.2). The improved dispersion afforded by the PEG coating increases the number of such sites. In our previous study on unmodified ZnO-filled PLA/PBS/CF composites, the highest SE_T_ of 85 dB required a loading of 7 wt % [14]. The present system therefore exceeds that value at approximately one-fifth of the nanoparticle content.

At 2 wt % PZ, SE_T_ decreases to 67 dB. Reflection is unchanged (R ≈ 0.69, SE_R_ ≈ 5 dB), so the loss of shielding arises from absorption, which falls from about 84 to 61 dB. SEM does not show micron-scale clusters at this loading, but the nodular features are more closely packed than at lower loadings (Figure 2h), and aggregation below the resolution and sampling of the micrographs cannot be excluded. Two contributions are consistent with the data: more frequent particle–particle contact, which reduces the interfacial area available for polarization, and a larger total amount of insulating PEG at particle–fiber junctions, which can weaken contact-mediated loss. The simultaneous decrease in strength and strain from 1.5 to 2 wt % supports a change in particle packing at this loading. The present data do not separate the two contributions.

Comparable losses of EMI performance beyond an optimal filler ratio have been reported and attributed to nanoparticle agglomeration [7,8,41]. A loading of 1.5 wt % PZ therefore represents the optimum for shielding, providing the best balance between dispersion quality, the number of polarizable ZnO–fiber junctions, preservation of the CF conductive network and impedance matching.

**Figure 5:**
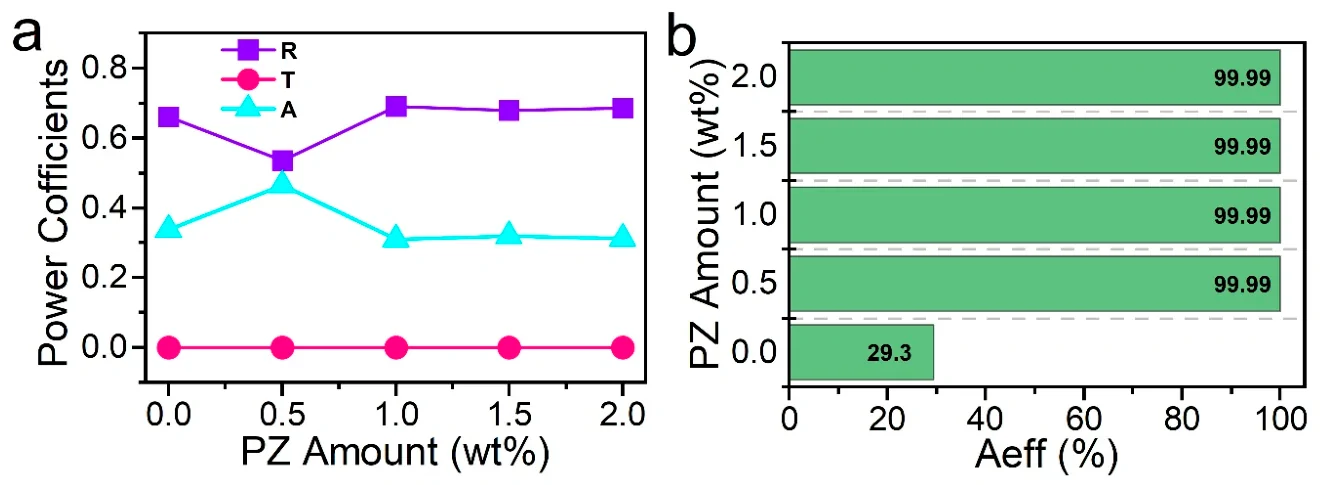
Power coefficients as a function of (**a**) PZ content and (**b**) effective absorbance, A_eff_, of the composites at 10 GHz.

The influence of PZ content on the reflectance (R), absorbance (A) and transmittance (T) power coefficients is presented in Figure 5a. The transmittance is essentially zero for every sample, showing that almost all incident radiation is either attenuated within the material or reflected at the surface, and that wave transmission is suppressed to a negligible level. This is consistent with the high SE_T_ values reported above, since high total shielding is intrinsically linked to low transmission.

For the PLA/PBS/CF composite the reflectance coefficient is approximately 0.66, decreasing to about 0.53 upon the addition of 0.5 wt % PZ. This reduction indicates that the surface impedance of the composite approaches that of free space, which reduces reflection at the surface and facilitates the entry of waves into the material. A low reflection coefficient is a characteristic feature of absorption-dominant EMI materials [9,57]. Correspondingly, the absorption coefficient increases from approximately 0.34 to 0.47, indicating that PZ nanoparticles substantially enhance the dissipation of electromagnetic energy. Increasing the PZ content while maintaining good filler dispersion increases the number of ZnO–CF contacts and fiber junctions bridged by ZnO, at which polarization loss can operate in the X-band (Section 3.5.2) [56].

At 1 wt % PZ and above, R returns to approximately 0.68–0.69 while A settles near 0.31–0.32. This may appear inconsistent with the high SE_A_ and SE_T_ values recorded for the 1 and 1.5 wt % samples, but the power coefficients express only the partitioning of incident energy and not the absolute attenuation capacity in decibels. A high reflection coefficient is therefore not necessarily associated with low shielding: as the conductive network formed by the carbon fibers becomes denser, waves may be reflected more strongly at the surface while the fraction that enters the material is attenuated far more effectively. In composites containing 1–1.5 wt % PZ, the strong conductive network derived from CF and the additional polarization loss introduced by ZnO act in combination to improve overall performance. It has been emphasized that both absorption and reflection mechanisms can contribute concurrently in hybrid conductor–dielectric filler systems, and that optimal EMI performance often results from a compromise between them [6].

The effective absorbance, A_eff_, shown in Figure 5b, quantifies the fraction of the non-reflected energy that is absorbed and is the appropriate metric for determining whether shielding is absorption weighted. The unfilled PLA/PBS blend exhibits an A_eff_ value of only 29.3%, indicating that it cannot efficiently absorb the electromagnetic energy reaching it. The A_eff_ value increases to 99.84% on adding 20 wt % CF [14]. All composites containing 0.5, 1, 1.5 and 2 wt % PZ reach A_eff_ values of 99.99%, demonstrating that essentially all the energy entering the material without being reflected at the surface is converted into thermal energy. Values of A_eff_ above 90% are considered indicative of excellent absorption-controlled EMI shielding [9]. This result confirms that the addition of CF and PEG-coated ZnO converts the shielding mechanism from a reflection-weighted to a strongly absorption-dominant one, in full agreement with the SE_A_ and SE_T_ findings.

#### 3.5.2. Loss Mechanisms in the X-Band

The relaxation frequency of Maxwell–Wagner–Sillars (MWS) interfacial polarization is governed by the conductivity of the phases that form the interface [51]. For dilute spherical particles of conductivity σ_p_ and permittivity ε_p_ in an insulating matrix of permittivity ε_m_, the classical Maxwell–Wagner expression gives f_MWS_ ≈ σ_p_/[2πε_0_(2ε_m_ + ε_p_)]. With ε_m_ ≈ 3 for the polyester matrix and ε_p_ ≈ 8–9 for ZnO, a particle conductivity of 10^−2^ S m^−1^ places the relaxation near 10 MHz, and a relaxation within the X-band would require a conductivity of the order of 10 S m^−1^. The conductivity of the ZnO used here was not measured. For interfaces between the insulating matrix and poorly conducting ZnO particles (σ_p_ ≪ 1 S m^−1^), the MWS relaxation therefore lies at MHz frequencies or below and is not expected to contribute significantly to loss at 8–12 GHz. In the discussion that follows, interfacial polarization is considered only where the conductive fiber network is involved.

Three loss processes are more likely to operate in the X-band. The first is conduction loss in the percolated carbon-fiber network, which underlies the rise in SE_T_ from 1.7 dB for the PLA/PBS blend to 30.7 dB on adding 20 wt % CF [14]. The second is polarization at interfaces that involve this network, such as fiber–matrix boundaries and junctions between fibers bridged by ZnO particles. At such junctions, the local effective conductivity is set by contact and tunnelling resistances and is broadly distributed, so part of the resulting relaxation spectrum can fall in the GHz range. The third is dipolar polarization at localized charge sites. Polarization loss arising from localized electrons at atomic-scale sites has been reported in the microwave range for Ni single-atom absorbers, where it was linked to unpaired Ni 3d electrons [58]. In ZnO, oxygen vacancies and zinc interstitials act as such sites, and defect-induced dipole polarization has been identified as a contributor to microwave loss in ZnO-based absorbers [59]. The defect population of the ZnO used in this work was not characterized.

The PZ content of the composites corresponds to only about 0.1–0.4 vol %, estimated from the compositions in Table 2 and nominal densities. At such low volume fractions, the intrinsic dielectric loss of the particles is unlikely to account on its own for the increase in SE_A_ from about 25 dB for PLA/PBS/CF to about 84 dB at 1.5 wt % PZ (Figure 4b,d). The effect of PZ is more plausibly exerted through the fiber network, by adding polarizable junctions at fiber–fiber and fiber–matrix contacts, which corresponds to the second process above. This assignment is qualitative, because the complex permittivity (ε′, ε″) and the DC conductivity of the composites were not measured, and separating the conduction and polarization contributions would require these data.

### 3.6. Benchmarking Against Reported Polymer EMI Shielding Materials

Table 6 places the present composites in the context of recently reported polymer-based EMI shielding materials operating in the X-band. The SE_T_ values achieved here (43–89 dB) exceed those of most biodegradable and non-biodegradable polymer composites reported to date, and the maximum of 89 dB is obtained at a total filler content of 21.5 wt %, of which only 1.5 wt % is nanoparticulate. Two features distinguish this system. The first is filler efficiency: comparable or higher shielding is achieved at nanoparticle loadings an order of magnitude below those used in ZnO-based systems such as ZnO-decorated carbonated cotton fiber. The second is the combination of ultra-high shielding with retained mechanical integrity, which the unmodified-ZnO analogue does not provide at any loading.

The comparison with unmodified ZnO in the same matrix [14] is the most direct. Unmodified ZnO required 7 wt % to reach its highest SE_T_ of 85 dB, at which loading the composite failed at 0.06% strain. PEG-coated ZnO reached a comparable value, 89 dB, at 1.5 wt %, about one-fifth of that loading, while retaining 2.19% strain.

## 4. Conclusions

This study demonstrates that an adsorbed poly(ethylene glycol) layer on ZnO nanoparticles improves the dispersion limitation constraining biodegradable PLA/PBS/CF EMI shielding composites, at a nanoparticle loading far below that previously required.

FTIR supports PEG adsorption. The ether C–O–C band shifts by +14 cm^−1^ (1093 → 1107 cm^−1^) and the multi-component envelope characteristic of bulk semicrystalline PEG collapses to a single broadened band, both of which require the chains to be in an adsorbed rather than a bulk environment.

Dispersion changes markedly with the coating. At a matched 1 wt % loading, unmodified ZnO accumulates in dense bands along the fiber–matrix interface, whereas coated particles remain uniformly distributed with no compact clusters up to 2 wt %. This translates directly into mechanical performance: at 1 wt % the coated system delivers 42% higher strain, 38% higher strength and 13% higher modulus than its unmodified counterpart, and 1PZ-CF is the only composition in either system to exceed the strength of the unfilled CF reference while also improving ductility. The catastrophic embrittlement of the unmodified system (0.06% strain at break at 7 wt %) is absent; no PZ composition falls below 1.92%. PEG is present in quantities far too small to plasticize the matrix and no T_g_ depression is observed. The improvement is therefore attributed to improved dispersion combined with a localized compliant interphase at the particle surface.

X-band shielding reached 89 dB at 1.5 wt % PZ, comparable to the 85 dB obtained with 7 wt % unmodified ZnO, that is, at about one-fifth of the nanoparticle loading and with 2.19% instead of 0.06% strain. The decline to 67 dB at 2 wt % defines the practical upper bound. Because 1 wt % PZ already provides 71 dB, which is well above the 20 to 30 dB commercial threshold, it is suitable when structural integrity is the priority, while 1.5 wt % can be used when maximum shielding is required. Surface passivation of the dielectric nanofiller is therefore a practical design route for shielding composites based on biodegradable matrices.

## Figures and Tables

**Table 1:** Materials used in this study.

Material	Grade/Specification	Supplier
Poly(lactic acid) (PLA)	Ingeo 2003D; Mw: 120,000 g mol^−1^; density 1.24 g cm^−3^; T_m_ ≈ 155 °C	NatureWorks (Minnetonka, MN, USA)
Poly(butylene succinate) (PBS)	BioPBS™ FZ71PM; Mw: 104,000 g mol^−1^; density 1.26 g cm^−3^; T_m_ ≈ 115 °C	PTT MCC Biochem (Bangkok, Thailand)
Carbon fiber (CF)	PA-sized chopped CF; bulk density 575 g L^−1^	DowAksa (Yalova, Türkiye)
ZnO nanoparticles	Nominal primary size 18 nm; purity 99.5%	Nanografi (Ankara, Türkiye)
Poly(ethylene glycol) (PEG)	M_v_ = 8000 g mol^−1^ (8 kDa)	—
Ethanol	Analytical grade, ≥99.5%	—

**Table 2:** Composite compositions and sample codes.

Sample Code	PLA/PBS (wt %)	CF (wt %)	PZ (wt %)
PLA/PBS	80/20	0	0
PLA/PBS/CF	80/20	20	0
0.5PZ-CF	80/20	20	0.5
1PZ-CF	80/20	20	1
1.5PZ-CF	80/20	20	1.5
2PZ-CF	80/20	20	2

**Table 3:** Tensile properties of PLA/PBS, PLA/PBS/CF and the PZ-containing composites. Values are means of four specimens ± one standard error.

Sample	Tensile Strain (%)	Tensile Strength (MPa)	Modulus (MPa)
PLA/PBS	9.33 ± 1.03	23.66 ± 2.11	4252 ± 971
PLA/PBS/CF	1.31 ± 0.11	70.75 ± 3.79	17,203 ± 2784
0.5PZ-CF	3.09 ± 0.23	59.35 ± 6.77	8549 ± 1371
1PZ-CF	2.35 ± 0.17	76.30 ± 1.75	14,939 ± 1540
1.5PZ-CF	2.19 ± 0.24	73.16 ± 3.16	13,348 ± 1771
2PZ-CF	1.92 ± 0.17	65.09 ± 2.61	13,467 ± 1042

**Table 4:** Thermal transitions of the samples obtained from DSC second heating curves.

Sample	T_g_−PLA (°C)	T_cc_ (°C)	T_m_−PBS (°C)	ΔH_m_−PBS (J/g)	T_m_−PLA (°C)	ΔH_m_−PLA (J/g)	X_c_−PLA (%)
PLA/PBS	55.3	107.8	118.0	16.6	158.5	20.3	27.3
PLA/PBS/CF	57.2	109.0	115.6	0.8	156.8	23.1	38.8
0.5PZ-CF	56.5	104.6	113.4	10.1	153.7	22.4	37.9
1PZ-CF	57.8	104.0	109.2	6.2	154.0	21.3	36.2
1.5PZ-CF	57.2	105.6	114.4	6.8	155.1	20.2	34.6
2PZ-CF	56.0	108.2	114.2	2.9	154.7	19.7	33.9

**Table 5:** Thermal degradation properties of the samples.

Sample	T_d5_ (°C)	T_d50_ (°C)	T_max_−1 (°C)	T_max_−2 (°C)	Residual Weight at 600 °C (%)
PLA/PBS	318.7	359.1	359.8	395.9	1.6
PLA/PBS/CF	299.1	325.5	324.4	364.7	12.9
0.5PZ-CF	263.1	305.3	297.7	378.3	19.0
1PZ-CF	257.5	301.2	293.6	376.1	20.3
1.5PZ-CF	258.2	298.4	289.9	374.9	20.7
2PZ-CF	256.5	303.2	290.2	371.9	24.7

**Table 6:** Comparison of the EMI shielding performance of PLA/PBS/CF/PZ composites (this work) with recently reported polymer-based EMI shielding materials in the X-band.

Composite System	Filler(s)	Filler Loading	SE_T_ (dB)	Dominant Mechanism	Ref.
PLA/PBS/CF/PZ	CF + PEG-coated ZnO	20 wt % CF + 0.5–2 wt % PZ	43–89	Absorption (>90%)	This work
PLA/PBS/CF/ZnO	CF + ZnO	20 wt % CF + 1–7 wt % ZnO	70–85	Absorption (>80%)	[14]
PA11/PLA/CF	Carbon fibre	10–30 wt % CF	15–28	Reflection	[15]
PLA/SCF + CNT	Short CF + CNT	20 wt % CF + 1 wt % CNT	≈52	Absorption	[17]
PLA/PEG/MWCNT	MWCNT	4 wt %	≈42	Absorption	[60]
PLA/GNP	Graphene nanoplatelets	15 wt %	≈15	Reflection	[61]
PLA/CNT/MXene	CNT + Ti_3_C_2_T_x_ MXene	Various	≈39	Absorption	[62]
CF-ZnO/cotton	CF + ZnO	30–70 wt % ZnO	32–38	Absorption (>80%)	[63]
Fe@C–CF foam	CF + Fe nanoparticles	—	≈62	Absorption	[64]
PLA/CF foam	Carbon fibre	20 wt % CF	≈27–30	Absorption	[65]

## Data Availability

The original contributions presented in this study are included in the article and Supplementary Material. Further inquiries can be directed to the corresponding author.

## Supplementary Materials

The following supporting information can be downloaded at: https://www.mdpi.com/article/10.3390/polym18192388/s1, Figure S1: bright-field STEM images of neat and PEG-coated ZnO nanoparticles showing particle morphology and size; Figure S2: SEM micrographs comparing PLA/PBS/CF composites containing 1 wt % PEG-coated and 1 wt % unmodified ZnO; Figure S3: individual tensile stress–strain curves for all PZ compositions (four specimens per composition); Table S1: Composition and PEG content of the PEG-coated ZnO (PZ) composites; Table S2: Comparison of tensile properties with the unmodified-ZnO system.
