# Supplementary material for: PEG Surface Modification of ZnO Nanoparticles Improves Ductility and Absorption-Dominant EMI Shielding in PLA/PBS/Carbon Fiber Composites
Source: Polymers (Basel). 2026 Sep 30;18(19):2388. doi: 10.3390/polym18192388 (PMC13666652; doi:10.3390/polym18192388)
Supplement: Supplementary file 1 [file polymers-18-02388-s001.zip › polymers-4540239-supplementary.pdf]

Supplementary Materials

PEG Surface Modification of ZnO Nanoparticles Improves Ductility and Absorption-Dominant EMI Shielding in PLA/PBS/Carbon Fiber Composites

Necla Altin [1], Saeid Darvishi [1,*], Bedriye Ucpinar [2], Alp Oral Salman [3], Ayse Aytac [1,4,*]

[1] Department of Chemical Engineering, Faculty of Engineering, Kocaeli University, 41001 Kocaeli, Türkiye

[2] Department of Chemical Engineering, Faculty of Engineering, Yeditepe University, 34755 İstanbul, Türkiye

[3] Department of Electronics and Communication Engineering, Faculty of Engineering, Kocaeli University, 41001 Kocaeli, Türkiye

[4] Department of Polymer Science and Technology, Kocaeli University, 41001 Kocaeli, Türkiye

[*] **Correspondence:** saeid.darvishi@kocaeli.edu.tr (S.D.); aaytac@kocaeli.edu.tr (A.A.)

Contents

Table S1. Composition and PEG content of the PEG-coated ZnO (PZ) composites.

Figure S1. Bright-field STEM images of neat and PEG-coated ZnO nanoparticles, showing particle morphology and size.

Figure S2. SEM micrographs of composites containing PEG-coated and unmodified ZnO nanoparticles.

Figure S3. Tensile stress–strain curves of the PZ-containing composites.

Table S2. Comparison of tensile properties with the unmodified-ZnO system.

The PEG loading used in the coating procedure was 10 wt % relative to ZnO, so that the coated particles (PZ) contain approximately 9.1 wt % organic phase. Table S1 gives the resulting PEG content of each composite. These values are the basis of the argument in Section 3.3.4 of the main text that the amount of PEG present is too small by one to two orders of magnitude to plasticize the PLA/PBS matrix in bulk.

Table S1. Composition and calculated PEG content of the PZ composites.

Sample code	CF (wt %)	PZ (wt %)	ZnO in composite (wt %)	PEG in composite (wt %)	PEG relative to polymer phase (wt %)
PLA/PBS	0	0	0	0	0
PLA/PBS/CF	20	0	0	0	0
0.5PZ-CF	20	0.5	0.455	0.045	0.057
1PZ-CF	20	1.0	0.909	0.091	0.115
1.5PZ-CF	20	1.5	1.364	0.136	0.174
2PZ-CF	20	2.0	1.818	0.182	0.233

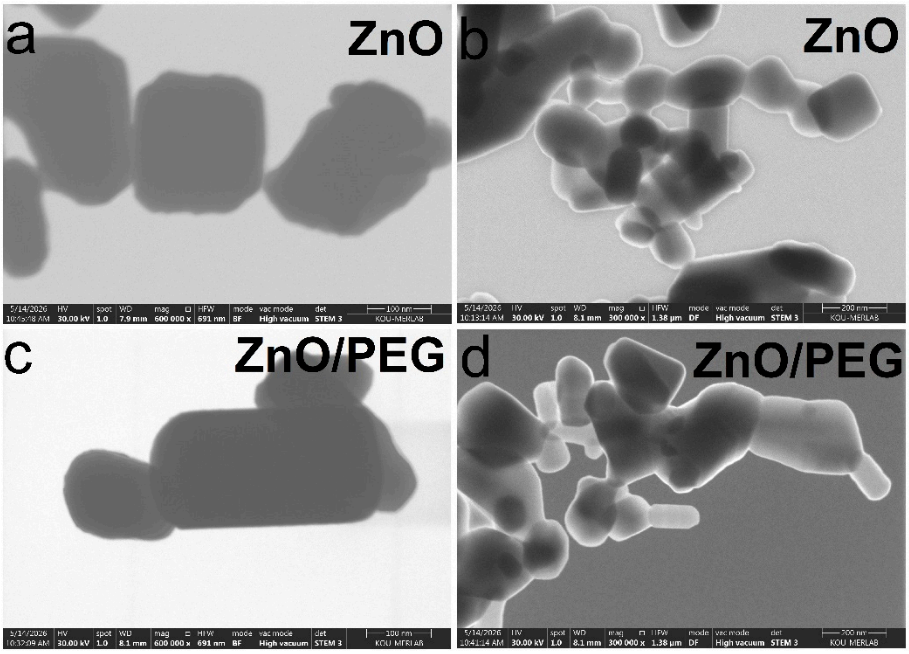

Figure S1. Bright-field STEM images of (a, b) as-received ZnO nanoparticles and (c, d) PEG-coated ZnO nanoparticles. Both powders consist of faceted, equiaxed particles of roughly 50–300 nm, considerably larger than the nominal 18 nm primary crystallite size quoted by the supplier, indicating that the as-received material is supplied as sintered polycrystalline aggregates. The adsorbed organic layer is at or below the resolution limit of the instrument and

no layer thickness is measured from these images. The coating is established by FTIR (Figure 1a of the main text). Scale bars: 100 nm (a, c), 200 nm (b, d).

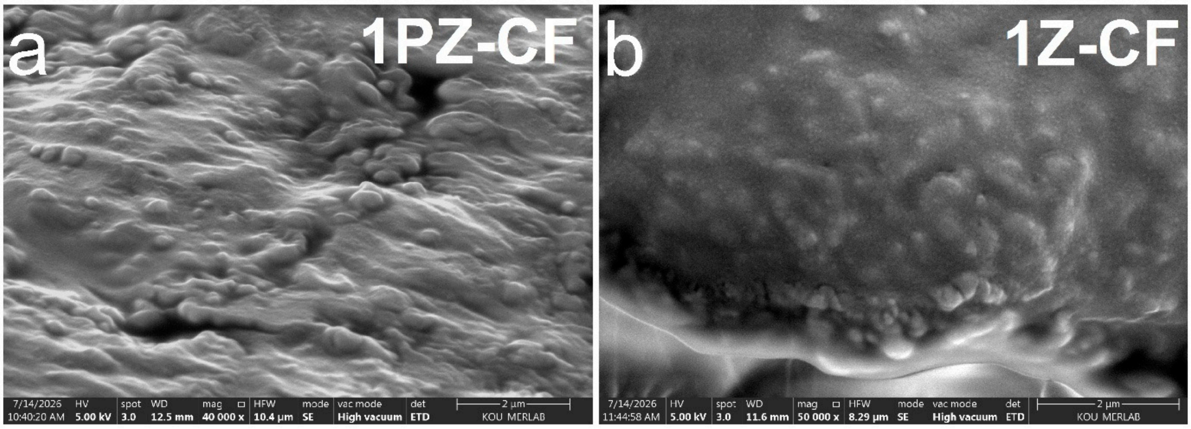

Figure S2. SEM micrographs of the fracture surfaces of PLA/PBS/CF composites containing (a) 1 wt % PEG-coated ZnO (1PZ-CF, ×40,000) and (b) 1 wt % unmodified ZnO (1Z-CF, ×50,000). The two composites are identical in matrix composition, fiber content and processing history and differ only in the surface state of the nanoparticles. In (b) the nanoparticles accumulate in dense, contiguous bands along the fiber–matrix interface region, leaving adjacent matrix areas comparatively depleted; in (a) the nanoparticulate phase is distributed uniformly at sub-micron scale with no comparable accumulation. Scale bars: 2 μm.

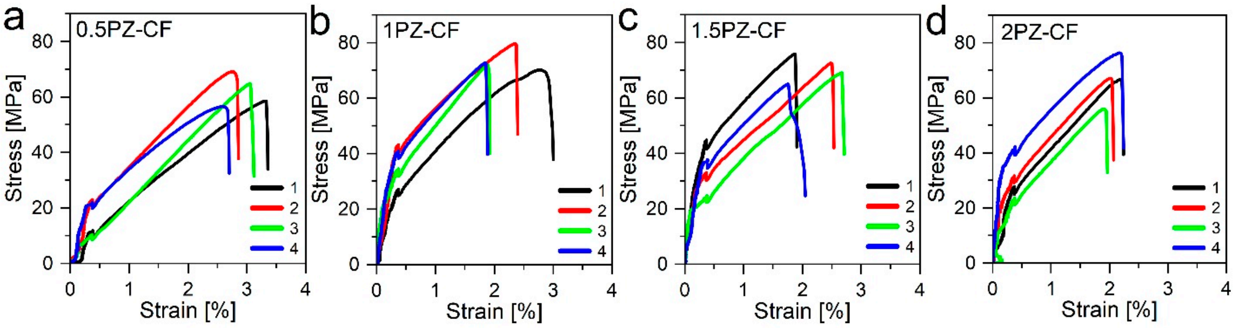

Figure S3. Tensile stress–strain curves of the PLA/PBS/CF composites containing (a) 0.5, (b) 1, (c) 1.5 and (d) 2 wt % PEG-coated ZnO. Four specimens were tested per composition, and all individual curves are shown. All compositions display a two-stage response: an initial linear region terminating in a small load discontinuity at approximately 0.3–0.4 % strain, attributed to the onset of localized fibre–matrix debonding, followed by an extended non-linear region in which the material continues to carry increasing load up to the maximum. The average parameters derived from these curves are given in Table 3 of the main text.

Table S2 compares the tensile properties of the PEG-coated ZnO (PZ) composites with those of the unmodified ZnO composites of identical matrix composition, fibre content and processing history, reported previously [1]. The PLA/PBS and PLA/PBS/CF reference values are common to both data sets, so the comparison is internally consistent. Values in parentheses are relative to the PLA/PBS/CF reference.

Table S2. Tensile properties of PEG-coated and unmodified ZnO composites relative to the PLA/PBS/CF reference.

Sample	Nanoparticle content (wt %)	Tensile strain (%)	Tensile strength (MPa)	Modulus (MPa)
PLA/PBS	—	9.33 (+612 %)	23.66 (−67 %)	4252 (−75 %)
PLA/PBS/CF	0	1.31 [1]	70.75 [1]	17,203 [1]
0.5PZ-CF	0.5	3.09 (+136 %)	59.35 (−16 %)	8549 (−50 %)
1PZ-CF	1.0	2.35 (+79 %)	76.30 (+8 %)	14,939 (−13 %)
1.5PZ-CF	1.5	2.19 (+67 %)	73.16 (+3 %)	13,348 (−22 %)
2PZ-CF	2.0	1.92 (+47 %)	65.09 (−8 %)	13,467 (−22 %)
1ZnO-CF [1]	1	1.66 (+27 %)	55.17 (−22 %)	13,274 (−23 %)
3ZnO-CF [1]	3	0.30 (−77 %)	56.68 (−20 %)	14,253 (−17 %)
5ZnO-CF [1]	5	0.18 (−86 %)	42.70 (−40 %)	10,273 (−40 %)
7ZnO-CF [1]	7	0.06 (−95 %)	28.53 (−60 %)	n.d.

At a matched loading of 1 wt %, surface modification raises tensile strain by 42 %, strength by 38 %, and modulus by 13 %. Across the composition series, no unmodified-ZnO composite exceeds the strength of the PLA/PBS/CF reference and all compositions above 1 wt % lose more than 75 % of its strain capacity, whereas every PZ composite retains at least 47 % more strain than the reference, and two exceed its strength.

Reference

1. Altin, N., et al., *Synergistic EMI Shielding Efficiency of Biodegradable PLA/PBS-Based Composites Reinforced with Carbon Fibers and ZnO Nanoparticles.* ACS omega, 2026. **11**(23): p. 34599.
